# Normative age-related changes in resting-state EEG alpha power and theta/alpha ratio across eyes-open and eyes-closed conditions

**DOI:** 10.3389/fnagi.2026.1869339

**Published:** 2026-07-28

**Authors:** Ye-Ji Park, Hoo-Joon Lee, Beom-Jin Kwon, Jin-Hyoung Jeong

**Affiliations:** Department of Biomedical Engineering, Catholic Kwandong University, Gangneung-si, Gangwon-Do, Republic of Korea

**Keywords:** alpha power, EEG, healthy aging, normative analysis, resting-state, theta/alpha ratio

## Abstract

Resting-state electroencephalography (EEG) provides a sensitive measure of large-scale neural dynamics and has been widely used to characterize age-related changes in brain function. However, many prior studies have relied on relatively small samples or single-condition recordings, limiting the generalizability of findings. In this study, we investigated normative age-related trajectories of EEG spectral features across eyes-closed (EC) and eyes-open (EO) conditions in a large cohort of healthy adults. After reproducible preprocessing and artifact rejection, a total of 3,229 resting-state EEG recordings from 608 participants aged 20–70 years were included. EEG data were analyzed across eyes-closed (EC) and eyes-open (EO) conditions, pre- and post-task timepoints, and baseline and follow-up sessions when available. We focused on occipital alpha relative power, global theta/alpha ratio, and occipital alpha peak frequency as spectral markers. Linear mixed-effects models with participant-specific random intercepts were used to account for repeated measurements, and model estimates were reported with standard errors, confidence intervals, test statistics, and false discovery rate-adjusted *p*-values. Results showed a robust age-associated decrease in occipital alpha relative power, with lower alpha expression in EO than EC. In contrast, the global theta/alpha ratio increased with age, suggesting an age-associated shift in conventional spectral balance toward relatively greater low-frequency predominance. Occipital alpha peak frequency showed weaker and more variable patterns and was therefore interpreted as a supplementary marker. These findings provide normative descriptive information on age-associated resting-state EEG spectral variation in healthy adults and suggest that EC and EO recordings may be more informatively analyzed as distinct recording contexts rather than assumed to provide fully interchangeable baseline estimates.

## 1 Introduction

Resting-state electroencephalography (EEG) is a representative non-invasive method for measuring spontaneous neural activity in the absence of externally imposed tasks, and it has been widely applied in studies of cognition, arousal, psychiatric disorders, and normal aging (Hashemi et al., 2016; Scally et al., 2018; Vlahou et al., 2014). Because EEG provides high temporal resolution and can capture large-scale oscillatory dynamics under relatively simple recording conditions, it is particularly well-suited for examining how brain function changes across adulthood (Hashemi et al., 2016; Scally et al., 2018; Vlahou et al., 2014). Healthy aging is not limited to overt cognitive decline or clinical disease, but also involves gradual changes in neural synchronization, cortical excitability, sensory processing efficiency, and the organization of large-scale brain rhythms (Donoghue et al., 2020; Knyazeva et al., 2018; Park et al., 2024; Scally et al., 2018; Vlahou et al., 2014). Accordingly, resting-state EEG can provide physiologically meaningful information for describing age-associated spectral variation in healthy adulthood (Donoghue et al., 2020; Hashemi et al., 2016; Knyazeva et al., 2018; Park et al., 2024; Scally et al., 2018).

Among resting-state EEG findings, age-related changes in alpha-band activity have been reported most consistently. Alpha rhythms are typically most prominent during rest, especially under eyes-closed conditions, and have been linked to visual disengagement, cortical inhibition, attentional allocation, and thalamocortical synchronization (Barry and De Blasio, 2017; Barry et al., 2007; Grandy et al., 2013; Klimesch, 1999; Trammell et al., 2017). Previous studies have shown that alpha power tends to decrease with increasing age, and that alpha peak frequency may also slow across adulthood, suggesting age-associated changes in dominant alpha characteristics (Angelakis et al., 2004; Barry and De Blasio, 2017; Cragg et al., 2011; Finnigan and Robertson, 2011; Grandy et al., 2013; Knyazeva et al., 2018; Park et al., 2024; Posthuma et al., 2001; Scally et al., 2018). In parallel, aging has also been associated with changes in lower-frequency activity and with ratio-based metrics reflecting a relative shift in conventional spectral balance toward lower-frequency predominance (Prichep, 2005; Rodriguez et al., 1999; Trammell et al., 2017). These spectral changes have often been discussed in relation not only to pathological aging and cognitive impairment but also to normative aging processes observed in healthy adults (Knyazeva et al., 2018; Prichep, 2005; Rodriguez et al., 1999; Rossini et al., 2007).

Despite these findings, several limitations remain in the existing literature. First, many studies have relied on relatively small samples or age-restricted cohorts, limiting the stability and generalizability of observed age effects (Finnigan and Robertson, 2011; Knyazeva et al., 2018; Scally et al., 2018). Second, age ranges have often been unevenly distributed across groups, making it difficult to characterize continuous age-related trajectories across adulthood (Finnigan and Robertson, 2011; Hashemi et al., 2016; Scally et al., 2018). Third, although recording condition strongly influences resting-state EEG, many prior studies have examined only one condition, either eyes-closed (EC) or eyes-open (EO), rather than directly considering both within the same analytic framework (Barry and De Blasio, 2017; Barry et al., 2007; Jabès et al., 2021; Marx et al., 2004; Rihs et al., 2007).

This issue is especially important because EC and EO may not represent fully equivalent resting-state recording contexts, particularly when conventional spectral summary measures are used to characterize age-associated EEG variation. Under EC, posterior alpha activity is typically enhanced due to the absence of visual input and the relative stabilization of internally oriented brain states. Under EO, alpha power is attenuated as visual monitoring and environmental engagement increase, and the spectral topography may become comparatively desynchronized (Barry and De Blasio, 2017; Barry et al., 2007; Jabès et al., 2021; Marx et al., 2004; Rihs et al., 2007). Thus, even within the same individual, EC and EO conditions can influence the magnitude and distribution of resting-state EEG spectral features. Examining both conditions within the same analytic framework may therefore help clarify whether age-associated EEG spectral patterns are consistently observed across recording states or partly shaped by eye-state context (Jabès et al., 2021; Marx et al., 2004; Rihs et al., 2007).

Another unresolved issue concerns which EEG spectral markers are most robust and interpretable in normative aging research. Alpha peak frequency is often considered a meaningful index related to cognitive efficiency, arousal, and individual differences in neural processing, but in practice its estimation may become unstable when the alpha peak is weak or poorly defined (Angelakis et al., 2004; Cragg et al., 2011; Doppelmayr et al., 1998; Finnigan and Robertson, 2011; Grandy et al., 2013; Klimesch, 2012; Posthuma et al., 2001). By contrast, relative alpha power and ratio-based indices such as the theta/alpha ratio may offer more stable and interpretable summaries of age-related spectral change (Prichep, 2005; Rodriguez et al., 1999; Trammell et al., 2017). Therefore, it is useful to evaluate multiple candidate markers simultaneously in order to identify which EEG indices most reliably capture normative aging-related changes in healthy adults.

Against this background, the present study aimed to characterize age-associated changes in resting-state EEG spectral markers using a large open-access dataset of healthy adults. The primary contribution of this study is not to propose a previously unknown direction of EEG aging change, but rather to provide a large-sample and condition-sensitive characterization of established resting-state EEG aging markers across both EC and EO states. We focused on occipital alpha relative power and the global theta/alpha ratio as primary markers, with occipital alpha peak frequency examined as a supplementary marker. By modeling repeated recordings within participants, this study sought to clarify whether age-associated reductions in posterior alpha activity and increases in theta/alpha ratio are consistently expressed across resting-state conditions or are partly dependent on eye-state context. We hypothesized that increasing age would be associated with lower occipital alpha relative power and higher global theta/alpha ratio, consistent with age-associated spectral slowing. We further expected these patterns to be observable across both EC and EO conditions, while differing in magnitude according to eye state. Occipital alpha peak frequency was examined as a supplementary marker because peak-based estimates may be less stable when the alpha peak is weak or poorly defined.

## 2 Materials and methods

### 2.1 Study design and dataset

This study was a secondary analysis of the Dortmund Vital Study resting-state EEG dataset, publicly available through OpenNeuro as ds005385. The dataset includes healthy adult participants aged 20–70 years and contains repeated resting-state EEG recordings acquired under both eyes-closed (EC) and eyes-open (EO) conditions. Recordings were obtained at pre- and post-task timepoints, and follow-up sessions were available for a subset of participants. The original resting-state EEG protocol included approximately 3 min of EC recording followed by approximately 3 min of EO recording.

EEG was recorded using a 64-channel elastic cap arranged according to the international 10–20 electrode placement system. The recordings were acquired using a BrainVision BrainAmp DC system with FCz as the online reference and a sampling rate of 1,000 Hz. The original recording protocol used an online low-pass filter of 250 Hz, and electrode impedances were kept below 10 kΩ when possible.

The final analysis included 3,229 EEG recordings from 608 healthy adults. Participants ranged in age from 20 to 70 years. Both EC and EO recordings were available for the same individuals, and repeated measurements were available across pre/post timepoints and baseline/follow-up sessions for some participants. Because multiple recordings from the same participant are not statistically independent, mixed-effects models with participant-specific random intercepts were used as the primary inferential framework.

### 2.2 EEG preprocessing

Electroencephalography preprocessing was conducted using a standardized Python-based workflow. First, the continuous EEG recordings were loaded from the original EDF files. A 50 Hz notch filter was applied to reduce line noise, followed by band-pass filtering from 1 to 45 Hz to remove slow drifts and high-frequency noise. The data were then re-referenced to the average reference.

The continuous EEG signals were segmented into fixed-length 2-s epochs. Epochs were rejected when the peak-to-peak amplitude exceeded 150 μV, which was used as an automated threshold to remove epochs contaminated by large-amplitude artifacts such as eye blinks, movement artifacts, or muscle activity. Recordings with fewer than five retained clean epochs after artifact rejection were excluded from the final analysis. The number of retained epochs was stored for each recording and used to monitor data quality. All spectral features were extracted only from artifact-cleaned epochs.

No manual artifact rejection was performed in the present pipeline. Independent component analysis was not applied; therefore, residual ocular or muscle artifacts cannot be fully excluded. This limitation is acknowledged in the Discussion.

### 2.3 Spectral feature extraction

Power spectral density (PSD) was estimated from the artifact-cleaned 2-s epochs using Welch’s method. Spectral estimates were computed over the 1–30 Hz frequency range. The frequency resolution of the extracted spectral features was approximately 0.9766 Hz. Frequency bands were defined as follows: delta, 1–4 Hz; theta, 4–8 Hz; alpha, 8–13 Hz; and beta, 13–30 Hz.

Occipital alpha relative power was calculated from the occipital electrode cluster consisting of O1, Oz, and O2. For each recording, alpha-band power was averaged across the occipital cluster and expressed relative to total power in the 1–30 Hz range. This measure was used to quantify posterior alpha dominance while reducing the influence of interindividual differences in overall EEG amplitude.

The global theta/alpha ratio was calculated by dividing global theta-band power by global alpha-band power. Global values were computed by averaging across all available scalp channels after preprocessing and artifact rejection. This ratio was used as an interpretable summary of the relative predominance of slower theta activity over alpha activity.

Occipital alpha peak frequency was extracted as a supplementary marker. For each recording, the mean occipital PSD was calculated from O1, Oz, and O2, and the alpha peak frequency was identified within the predefined 8–13 Hz alpha range. No additional smoothing procedure beyond the Welch PSD estimation step was applied. The peak was selected as the maximum PSD value within the 8–13 Hz occipital alpha range. When no clearly isolated alpha peak was present, the maximum within this range was still retained to avoid introducing an additional subjective exclusion criterion, but the resulting estimate was interpreted cautiously. No participant-level exclusion was applied solely on the basis of weak or absent alpha peaks. For this reason, alpha peak frequency was treated as a supplementary outcome rather than a primary marker.

### 2.4 Statistical analysis

Descriptive statistics were calculated at both the participant and recording levels. Participant-level summaries included age, sex, and age-group distributions. Recording-level summaries included the number of EEG recordings across EC/EO conditions, pre/post timepoints, and baseline/follow-up sessions.

The primary inferential analyses used linear mixed-effects models to account for repeated measurements within participants. Each model included a participant-specific random intercept. Fixed effects included standardized age, quadratic age, eye state, timepoint, session, sex, the interaction between age and eye state, and the interaction between age and timepoint. The main outcomes were occipital alpha relative power and global theta/alpha ratio. Occipital alpha peak frequency was analyzed as a supplementary outcome.

Model results were reported as regression coefficients, standard errors, 95% confidence intervals, test statistics, nominal *p*-values, and false discovery rate-adjusted *p*-values. False discovery rate correction was applied across the tested fixed-effect terms within each outcome. Because the present study focused on a limited set of predefined spectral outcomes, correction was applied within each outcome model rather than across all possible EEG features; this scope of correction is acknowledged when interpreting the results. Locally weighted scatterplot smoothing (LOWESS) was used to visualize age-associated trajectories under EC and EO conditions. Additional robustness checks included winsorized sensitivity analyses and participant-level mean visualizations.

Because the present analysis was based on conventional band-power features, periodic and aperiodic spectral components were not separated. Therefore, the possible influence of aperiodic spectral background activity on band-power estimates is addressed as an important limitation.

## 3 Results

### 3.1 Sample characteristics

The final analysis included a total of 3,229 EEG recordings obtained from 608 healthy adults (Table 1). At the participant level, the mean age was 44.07 ± 14.51 years, with an age range of 20 to 70 years. There were 376 women (61.8%) and 232 men (38.2%). The age-group distribution was relatively balanced, consisting of 137 participants aged 20–29 years (22.5%), 111 aged 30–39 years (18.3%), 111 aged 40–49 years (18.3%), 140 aged 50–59 years (23.0%), and 109 aged 60 years or older (17.9%).

**TABLE 1:** Demographic characteristics of participants and distribution of electroencephalography (EEG) recordings across experimental conditions.

Section	Variable	Value
Participants	Total number of participants	608
	Age, mean ± SD	44.07 ± 14.51
	Age range	20–70 years
	Female	376 (61.8%)
	Male	232 (38.2%)
	Age group, 20–29 years	137 (22.5%)
	Age group, 30–39 years	111 (18.3%)
	Age group, 40–49 years	111 (18.3%)
	Age group, 50–59 years	140 (23.0%)
	Age group, ≥60 years	109 (17.9%)
Recordings	Total EEG recordings	3,229
	Eyes-closed (EC)	1,625
	Eyes-open (EO)	1,604
	Pre-task	1,617
	Post-task	1,612
	Baseline session	2,408
	Follow-up session	821

At the recording level, 1,625 recordings were obtained under eyes-closed (EC) conditions and 1,604 under eyes-open (EO) conditions, indicating an almost balanced distribution. Similarly, 1,617 recordings were acquired at the pre-task timepoint and 1,612 at the post-task timepoint, while 2,408 recordings were from baseline sessions and 821 were from follow-up sessions. This distribution suggests that the dataset was not excessively skewed toward any particular condition and thus provided a favorable structure for comparing conditions and evaluating age-related trajectories.

### 3.2 Age-related patterns of occipital alpha relative power and theta/alpha ratio

**FIGURE 1:**
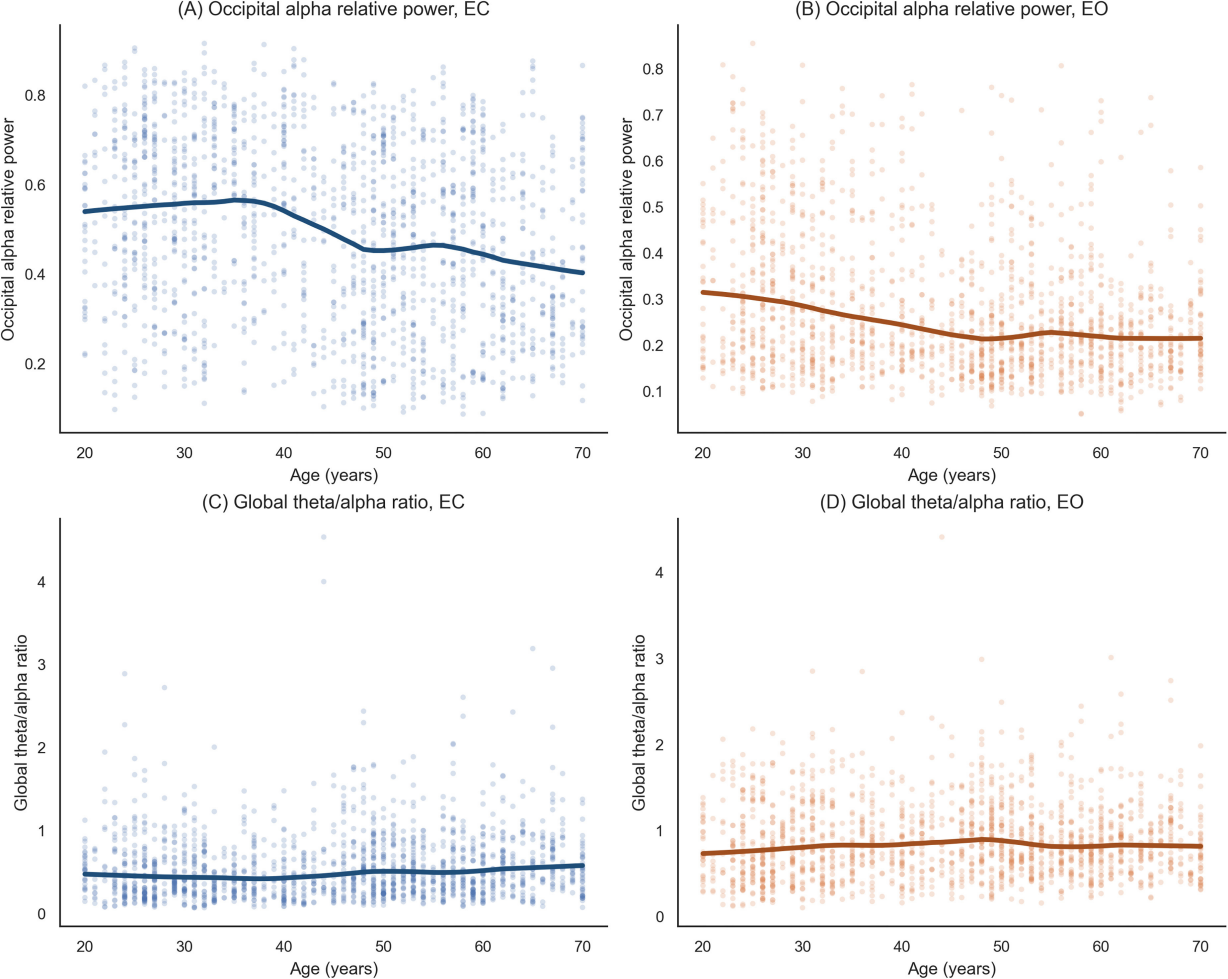
Age-associated trajectories of resting-state electroencephalography (EEG) spectral markers across eyes-closed and eyes-open conditions. **(A,B)** Show occipital alpha relative power as a function of age under eyes-closed and eyes-open conditions, respectively. **(C,D)** Show the global theta/alpha ratio as a function of age under eyes-closed and eyes-open conditions, respectively. Each point represents an EEG recording. Smoothed trajectories were estimated using locally weighted scatterplot smoothing (LOWESS) to visualize age-associated trends. The Figure is intended as a descriptive visualization of spectral variation across adulthood; statistical inference is based on the mixed-effects models reported in Table 2.

Figure 1 presents the age-related trajectories of occipital alpha relative power and the global theta/alpha ratio under eyes-closed and eyes-open conditions. As shown in Figures 1A, B, occipital alpha relative power showed an overall decline with increasing age under both EC and EO conditions. However, the absolute level differed by condition, such that occipital alpha relative power remained higher under EC than EO even within the same age range. In particular, the decline was more clearly and smoothly expressed under EC, illustrating a gradual weakening of posterior alpha dominance across adulthood. Under EO, the overall direction was similar, but because the absolute values were lower and the dispersion was relatively greater, the visual pattern appeared somewhat flatter than under EC.

By contrast, as shown in Figures 1C, D, the global theta/alpha ratio showed an age-related pattern opposite to that of occipital alpha relative power. The theta/alpha ratio increased overall with age, and this increase was more evident under EC. Under EO, the upward direction was preserved, although the trajectory appeared somewhat flatter or plateaued in later adulthood. These findings suggest that conventional resting-state EEG spectral composition shifts with age toward relatively greater low-frequency predominance and weaker alpha expression. However, because periodic and aperiodic spectral components were not separated in the present analysis, this pattern should be interpreted as a conventional spectral slowing pattern rather than as direct evidence of isolated oscillatory slowing.

The mixed-effects models were consistent with the visual trajectories (Table 2). For occipital alpha relative power, standardized age showed a significant negative association, indicating lower posterior alpha relative power with increasing age. EO recordings showed substantially lower occipital alpha relative power than EC recordings. Pre-task recordings also showed lower values than post-task recordings, and follow-up recordings showed slightly lower values than baseline recordings. The age-by-eye-state interaction was not statistically significant after false discovery rate correction, suggesting that the overall age-related decline in occipital alpha relative power was broadly preserved across EC and EO conditions.

**TABLE 2:** Mixed-effects model results for primary resting-state electroencephalography (EEG) spectral markers.

Outcome	Predictor	β	SE	95% CI	z	*P*	FDR-adjusted *p*
Occipital α RP	Sex, male	−0.0035	0.0122	−0.0273 to 0.0204	−0.285	0.776	0.776
Occipital α RP	EO vs. EC	−0.2197	0.0038	−0.2271 to −0.2124	−58.559	<0.001	<0.001
Occipital α RP	Pre vs. post	−0.0256	0.0038	−0.0330 to −0.0183	−6.827	<0.001	<0.001
Occipital α RP	Follow-up vs. baseline	−0.0115	0.0051	−0.0215 to −0.0014	−2.234	0.025	0.044
Occipital α RP	Age, z-score	−0.0537	0.0065	−0.0664 to −0.0411	−8.328	<0.001	<0.001
Occipital α RP	Age × EO	0.0071	0.0038	−0.0002 to 0.0145	1.903	0.057	0.091
Occipital α RP	Age × pre	0.0088	0.0037	0.0014 to 0.0161	2.340	0.019	0.036
Occipital α RP	Age^2^	0.0039	0.0067	−0.0092 to 0.0170	0.587	0.557	0.581
Global θ/α ratio	Sex, male	0.0757	0.0315	0.0141 to 0.1374	2.407	0.016	0.032
Global θ/α ratio	EO vs EC	0.3218	0.0089	0.3044 to 0.3392	36.291	<0.001	<0.001
Global θ/α ratio	Pre vs. post	0.0139	0.0089	−0.0035 to 0.0313	1.567	0.117	0.165
Global θ/α ratio	Follow-up vs. baseline	−0.0158	0.0122	−0.0397 to 0.0081	−1.299	0.194	0.259
Global θ/α ratio	Age, z-score	0.0457	0.0165	0.0135 to 0.0780	2.777	0.005	0.012
Global θ/α ratio	Age × EO	−0.0270	0.0089	−0.0443 to −0.0096	−3.043	0.002	0.006
Global θ/α ratio	Age × pre	−0.0080	0.0089	−0.0253 to 0.0094	−0.901	0.368	0.464
Global θ/α ratio	Age^2^	−0.0141	0.0172	−0.0479 to 0.0197	−0.819	0.413	0.495

α RP, alpha relative power; θ/α, theta/alpha; EC, eyes closed; EO, eyes open; FDR, false discovery rate. Values are from linear mixed-effects models with participant-specific random intercepts. Age was standardized before modeling. EC, post-task, baseline, and female were used as reference categories.

For the global theta/alpha ratio, standardized age showed a positive association, indicating an age-associated increase in relative theta predominance over alpha activity. EO recordings showed a significantly higher theta/alpha ratio than EC recordings. The age-by-eye-state interaction was significant, suggesting that the magnitude of the age-associated theta/alpha pattern differed between EC and EO conditions. Together, the regression results support a complementary pattern of reduced occipital alpha relative power and increased theta/alpha ratio with age.

### 3.3 Occipital alpha peak frequency as a supplementary marker

Occipital alpha peak frequency was examined as a supplementary marker. Compared with occipital alpha relative power and the global theta/alpha ratio, alpha peak frequency showed a less straightforward pattern. The model suggested an age-associated decrease in occipital alpha peak frequency, but the effect was accompanied by condition- and timepoint-related variability. In addition, alpha peak frequency is inherently sensitive to the clarity of the individual alpha peak, and estimates may be less reliable when the alpha rhythm is weak or broadly distributed. Therefore, although the alpha peak frequency results are consistent with the possibility of age-related slowing of the dominant alpha rhythm, they were interpreted more cautiously than the relative alpha power and theta/alpha ratio findings.

### 3.4 Additional robustness analyses

Additional sensitivity and supplementary analyses showed that the direction of the main findings presented in the text was generally preserved. LOWESS trajectories derived from the raw and winsorized data were similar, and participant-level mean visualizations also reproduced the main patterns of declining occipital alpha relative power and increasing theta/alpha ratio. These findings suggest that the core results were not driven solely by a small number of extreme observations or by the repeated-measurement structure of the dataset, but rather reflected relatively stable normative patterns observed throughout the data.

## 4 Discussion

The present study evaluated age-associated variation in resting-state EEG spectral markers under eyes-closed and eyes-open conditions using a large open dataset of healthy adults. The principal findings may be summarized in three points. First, occipital alpha relative power decreased overall with increasing age under both EC and EO conditions. Second, the global theta/alpha ratio showed the opposite pattern, increasing with age and suggesting a shift in conventional spectral balance toward relatively greater low-frequency predominance. Third, occipital alpha peak frequency showed weaker and less consistent patterns than the other two markers and was therefore more appropriately interpreted as a supplementary outcome. Taken together, these findings provide descriptive evidence that healthy adult aging is associated with reduced posterior alpha dominance and a complementary increase in relative low-frequency predominance (Finnigan and Robertson, 2011; Knyazeva et al., 2018; Park et al., 2024; Prichep, 2005; Rodriguez et al., 1999; Scally et al., 2018; Trammell et al., 2017).

Although the direction of the present findings is broadly consistent with previous resting-state EEG aging studies, the contribution of this study should be interpreted primarily at the methodological and normative-descriptive levels. Age-related alpha attenuation and increased theta/alpha ratio have been reported previously, but many earlier studies examined a single resting-state condition or did not explicitly model repeated recordings within the same analytic framework. The present study therefore adds value by characterizing these established spectral aging patterns across both EC and EO conditions in a relatively large healthy adult cohort while accounting for repeated measurements within participants. This condition-sensitive perspective is useful because EC and EO represent different resting-state recording contexts and may not provide fully interchangeable estimates when conventional spectral markers of aging are interpreted. Thus, the main contribution of the present work should be understood as a reproducible, large-sample normative characterization of established EEG aging patterns across recording conditions, rather than as evidence for a new mechanistic biomarker of neural aging.

The decline in occipital alpha relative power is one of the most interpretable findings in the context of aging. Alpha rhythms have long been linked to thalamocortical synchronization, cortical inhibition, visual disengagement, and attentional state regulation (Barry and De Blasio, 2017; Barry et al., 2007; Grandy et al., 2013; Klimesch, 1999; Trammell et al., 2017). Accordingly, a reduction in occipital alpha relative power with increasing age may reflect broader age-related changes in spontaneous neural coordination rather than a narrow reduction confined to a single frequency band (Angelakis et al., 2004; Barry and De Blasio, 2017; Cragg et al., 2011; Finnigan and Robertson, 2011; Grandy et al., 2013; Knyazeva et al., 2018; Park et al., 2024; Posthuma et al., 2001; Scally et al., 2018). Previous studies have likewise reported attenuation of alpha power or slowing of dominant alpha characteristics in older adults, and the current results are broadly consistent with that literature (Angelakis et al., 2004; Cragg et al., 2011; Finnigan and Robertson, 2011; Knyazeva et al., 2018; Park et al., 2024; Posthuma et al., 2001; Scally et al., 2018). Because the present analysis used relative occipital alpha power, the findings are particularly relevant to posterior alpha dominance as a physiologically meaningful resting-state feature of healthy adulthood.

The age-related increase in the theta/alpha ratio complements this interpretation from the opposite direction. If alpha activity decreases while lower-frequency activity becomes relatively more prominent, a ratio-based metric such as theta/alpha is expected to increase (Prichep, 2005; Rodriguez et al., 1999; Trammell et al., 2017). This pattern is consistent with the broader notion of EEG slowing frequently discussed in aging research, but in the present study it should be interpreted as a conventional spectral balance measure rather than as direct evidence of isolated oscillatory slowing, because periodic and aperiodic components were not separated (Prichep, 2005; Rodriguez et al., 1999; Rossini et al., 2007). Importantly, ratio-based measures may also be useful because they summarize relative spectral balance rather than isolated band-specific values, potentially improving interpretability when multiple components shift simultaneously with age (Prichep, 2005; Rodriguez et al., 1999). In this respect, the present findings suggest that occipital alpha relative power and theta/alpha ratio function as complementary markers that capture related aspects of normative aging from different spectral perspectives.

A notable contribution of this study is the joint consideration of EC and EO conditions. EC and EO are not merely technical recording variants; they represent different resting-state recording contexts that differ in visual input, cortical arousal, and posterior alpha expression (Barry and De Blasio, 2017; Barry et al., 2007; Jabès et al., 2021; Marx et al., 2004; Rihs et al., 2007). In the present study, occipital alpha relative power was consistently higher under EC than EO, while the overall age-associated downward trend was preserved across both conditions. Similarly, the theta/alpha ratio showed a general age-related increase across conditions, although the shape and clarity of the trajectories differed somewhat between EC and EO. These findings suggest that age-associated spectral variation is observable across resting-state contexts, while the magnitude and visual expression of this variation remain condition-dependent. This supports a cautious reporting strategy in which EC and EO are either analyzed separately or explicitly modeled as recording conditions, rather than being assumed to provide identical baseline estimates (Jabès et al., 2021; Marx et al., 2004; Rihs et al., 2007).

Occipital alpha peak frequency showed weaker and less stable results than the other markers. This does not mean that alpha peak frequency is physiologically unimportant. On the contrary, previous research has associated alpha peak frequency with arousal, processing efficiency, and cognitive ability, and age-related slowing of peak alpha frequency has been repeatedly reported (Angelakis et al., 2004; Cragg et al., 2011; Doppelmayr et al., 1998; Finnigan and Robertson, 2011; Grandy et al., 2013; Klimesch, 2012; Posthuma et al., 2001). However, alpha peak frequency estimation may become unstable when the alpha peak is not sharply defined, and this problem may be especially relevant in heterogeneous real-world datasets or in analyses spanning a broad adult age range (Angelakis et al., 2004; Cragg et al., 2011; Finnigan and Robertson, 2011; Grandy et al., 2013; Posthuma et al., 2001). In the present dataset, alpha peak frequency therefore appeared less robust as a normative summary marker than occipital alpha relative power or theta/alpha ratio. For this reason, it was treated as a supplementary rather than primary indicator in the present study.

The present study has several strengths. First, it examined a relatively large number of recordings obtained from healthy adults covering a broad age range, which improves the descriptive value of the observed trajectories (Babayan et al., 2019; Hashemi et al., 2016). Second, by jointly analyzing EC and EO conditions, the study was able to compare condition-specific expression and shared age-related directionality within the same dataset (Barry and De Blasio, 2017; Barry et al., 2007; Jabès et al., 2021; Marx et al., 2004; Rihs et al., 2007). Third, the analytic framework accounted for repeated measurements within participants, thereby making more appropriate use of the structure of the dataset than a purely cross-sectional recording-level approach (Bates et al., 2015; Mirman, 2014). Together, these features strengthen the descriptive reliability of the observed age-associated spectral patterns while still requiring cautious interpretation as conventional EEG features rather than validated clinical biomarkers.

The present study focused on conventional spectral markers because they are interpretable, widely used, and suitable for large-scale normative description. However, contemporary EEG aging research increasingly incorporates source-level analyses, functional connectivity, graph-theoretical measures, and periodic/aperiodic spectral decomposition. These approaches may provide deeper mechanistic insight into whether age-associated EEG changes reflect altered oscillatory synchronization, changes in network organization, shifts in aperiodic background activity, or a combination of these processes. Therefore, the present findings should be viewed as descriptive spectral patterns rather than a complete mechanistic account of neural aging.

Several limitations should be considered.

First, although repeated measurements were available for some individuals, the present study primarily addressed age-associated variation across adulthood rather than strict within-person longitudinal aging trajectories. Accordingly, the results should be understood as age-associated cross-sectional spectral variation analyzed within a repeated-measurement framework, not as direct evidence of within-person longitudinal neural aging. Therefore, the findings should be interpreted as descriptive age-associated EEG patterns rather than direct evidence of individual aging trajectories.

Second, several potentially important confounding factors could not be fully incorporated into the present models. Education level, sleep status, psychoactive medication use, lifestyle factors, and cognitive reserve may all influence resting-state EEG activity and may vary systematically with age. In addition, detailed neuropsychological or cognitive performance measures were not analyzed in relation to the EEG markers. Therefore, the observed spectral changes should not be interpreted as direct clinical or cognitive markers of impairment.

Third, the preprocessing pipeline relied on automated amplitude-based artifact rejection and did not include manual artifact inspection or independent component analysis. Although this approach was suitable for large-scale processing, residual ocular or muscle artifacts may remain and should be considered when interpreting the findings.

Fourth, the present analysis did not separate periodic oscillatory activity from the aperiodic spectral background. Because the present study used conventional band-power-based spectral features, the observed age-associated decrease in alpha relative power and increase in theta/alpha ratio should not be interpreted as reflecting purely oscillatory changes. Age-related alterations in the aperiodic spectral background may also contribute to apparent band-power differences. Future studies should apply periodic/aperiodic decomposition approaches, such as the specparam framework, to determine whether the observed age-associated spectral patterns reflect changes in oscillatory alpha activity, aperiodic background activity, or both.

Fifth, Figure 1 was used as a descriptive visualization of age-associated spectral patterns based on LOWESS smoothing and does not include confidence bands around the fitted trajectories. Therefore, statistical uncertainty should be interpreted primarily from the mixed-effects model estimates and 95% confidence intervals reported in Table 2.

Despite these limitations, the present findings indicate that healthy aging is associated with interpretable and reproducible shifts in resting-state EEG spectral organization. In particular, reduced occipital alpha relative power and increased theta/alpha ratio appear to operate as complementary markers of healthy adult brain aging, whereas alpha peak frequency may serve as a useful but more variable supplementary marker. These findings may provide a normative reference point for future studies seeking to distinguish healthy aging from pathological aging, cognitive decline, or disorder-related EEG abnormalities (Rossini et al., 2007).

## 5 Conclusion

This study analyzed 3,229 resting-state EEG recordings from 608 healthy adults and examined age-associated spectral patterns across both EC and EO conditions. Occipital alpha relative power decreased with age, whereas the global theta/alpha ratio increased, suggesting a shift in conventional spectral balance toward relatively greater low-frequency predominance across adulthood. These patterns were observed while accounting for repeated measurements within participants and were expressed differently across EC and EO conditions. Occipital alpha peak frequency showed more variable results and should therefore be interpreted as a supplementary marker. Overall, the findings provide descriptive normative reference information for conventional resting-state EEG spectral features in healthy adults and support the value of reporting EC and EO conditions separately or explicitly modeling eye state when interpreting age-associated spectral patterns.

## Data Availability

Publicly available datasets were analyzed in this study. This data can be found here: Dortmund Vital Study resting-state EEG dataset, publicly available through OpenNeuro as ds005385. Further inquiries can be directed to the corresponding author.
